# Discitis in Children: A 10‐Year Retrospective Review in a Tertiary Pediatric Center

**DOI:** 10.1002/hsr2.70891

**Published:** 2025-06-11

**Authors:** Sara Farah, Seilesh Kadambari, Delane Shingadia

**Affiliations:** ^1^ Department of Paediatric Infectious Diseases and Immunology Great Ormond Street Hospital London UK; ^2^ University College London Great Ormond Street Institute of Child Health London UK

## 1 Introduction

Spondylodiscitis is infection of the intervertebral disc and adjacent vertebral bodies. It is rare in children, with an estimated incidence of 0.3 per 100.000 [1]. The most causative organisms are *Staphylococcus aureus* and *Kingella kingae* [2]. However, *Salmonella spp*. (typically in children with sickle cell disease), *Mycobacterium tuberculosis* (in high incidence regions), *Brucella spp* (with animal exposure and ingestion of unpasteurized dairy products) and fungal causes (in immunocompromisied hosts) can also cause disease [3].

The management is challenging, given the indolent clinical presentation, limited awareness amongst clinicians and paucity of epidemiological data to inform treatment guidelines. This study evaluated the clinical burden of spondylodiscitis at a single tertiary pediatric center.

## 2 Methods

We retrospectively reviewed patients with spondylodiscitis during January 2013 and December 2022 in our single centre. This study adhered to the Strengthening the Reporting of Observational Studies in Epidemiology reporting guideline for reporting observational studies (checklist attached). Patients were identified from hospital discharge data using ICD‐10 codes. The inclusion criteria were any child under 18 years old with clinical or radiographic features consistent with spondylodiscitis. Patients with granulomatous spondylodiscitis caused by mycobacterial disease were excluded. Electronic medical records were interrogated for demographic, clinical, laboratory, radiological and outcome data. Data were collected and descriptive analyses done using Microsoft Excel. This study received approval as a service evaluation (#3415) with the clinical audit/quality service at GOSH, in line with the criteria outlined by the National Health Service's Health Research Authority and adhering to the respective ethical guidelines.

## 3 Results

Nineteen patients with spondylodiscitis were identified and three had comorbidites (acute lymphoblastic leukemia, Mucopolysaccharidosis and epilepsy). None were secondary to any procedure (i.e., surgery, lumbar puncture or invasive procedure).

Children presented with nonspecific clinical features (i.e., gait disturbance, fever and localized pain) and moderately raised inflammatory markers (Table 1). An MRI spine was the most useful diagnostic tool at presentation and revealed pathological abnormalities in all those who had imaging. Of the 19 patients, 11 (57.9%) had blood cultures taken, with two patients (18.2%) having positive *staphylococcus aureus* and *bacillus cereus* cultures (both isolates fully sensitive). In total, 4/10 (40%) patients who had a spinal biopsy had a positive PCR result: *Candida tropicalis, Propionibacterium acnes, Aspergillus penicillioides*, and *Bacillus licheniformis*. The last two pathogens were treated as likely contaminants.

**Table 1:** Clinical characteristics of children with spondylodiscitis. Median results are displayed alongside interquartile ranges and number of patients with data.

	Complete recovery (*N* = 14, 73.7%)	Recovery with complication (*N* = 3, 15.8%)
Age < 2 years	10 (71.4%)	0 (0%)
Age 2–12 years (SD 1.0)	1 (7.1)%	1 (33.3%)
Age > 12 years (SD 2.1)	3 (21.4%)	2 (66.7%)
Male	7 (50%)	3 (100%)
Fever > 38℃	5 (35.7%)	1 (33.3%)
Back pain	5 (35.7%)	3 (100%)
Refusal to walk/sit/abnormal gait	8 (57.2%)	0 (0%)
Stiffness	12 (85.7%)	0 (0%)
Numbness	1 (7.1%)	0 (0%)
Weakness	1 (7.1%)	0 (0%)
Mean duration of symptoms before admission	31.8 days (3 – 84)	84 days (21 – 168)
Lumbar	12 (85.7%)	2 (66.7%)
Thoracic	1 (7.1%)	1 (33.3%)
Cervical	1 (7.1%)	0 (00%)
Mean CRP (mg/L)	26.3 (1‐ 92)	6 (1 − 112)
Mean WCC (10^9^/L)	10.5	10.6
Mean ESR (mm/hr)	53 (3 – 120)	12 (2‐160)
Positive blood culture	1 (10%)	1 (100%)
Biopsy obtained	7 (50%)	2 (66.6%)
Positive PCR	3 (42.9%)	0 (00%)
MRI at presentation: Signal change/enhancement	14 (100%)	3 (100%)
MRI at presentation: vertebral collapse	1 (7.1%)	3 (100%)
MRI at presentation: vertebral collection	6 (42.9%)	0 (0%)
MRI end of treatment: complete resolution	0 (0%)	0 (0%)
MRI end of treatment: residual damage	14 (100%)	3 (100%)
Mean duration IV antibiotics	4 weeks	7.2 weeks
Mean duration oral antibiotics	6 weeks	6 weeks

Overall, 14/19 (73.7%) patients had fully recovered, 3 (15.8%) recovered with complications, and 2 (10.5%) were lost to follow‐up. Older children were more likely to have complications albeit they were more likely to present later too. In the three patients who recovered with complications, the mean time taken to reach a diagnosis of spondylodiscitis was 84 days (SD 21.2) compared to 32 days (SD 6.1) in those who fully recovered. Children with complications required almost twice as long duration of IV antibiotics (7.2 weeks vs. 4 weeks) than those with full recovery.

In total, 9/15 (60%) of patients received ceftriaxone which was the most commonly used intravenous antibiotic. In half of patients, clindamycin was used as monotherapy or in combination with another antibiotic. Co‐amoxiclav was the most commonly used oral step down treatment. One patient received fluconazole for 3 months; they were immunocompromised and had *Candida tropicalis* isolated by PCR from the bone biopsy.

## 4 Discussion

This small study reveals a delay in making a diagnosis of spondylodiscitis can contribute to long term sequale. Our data highlight that young children presenting with persistent non specific back pain or gait disturbance should have a low threshold for evaluation of spondylodiscitis. This should include an MRI scan as part of the diagnostic work up.

In this study, we found no evidence to do a routine spinal biopsy. However, immunosuppressed children or those with persistent sypmptoms despite antibiotic treatment should warrant biopsy to rule out atypical infections and noninfective causes of disease.

To our knowledge, there are no consensus or published guidelines regarding the optimal treatment of SD in children. This in contrast to USA and European guidelines for the management of other osteoarticular infections in children [2, 4] and adult specific guidelines for the treatment of SD [5]. Our data, highlight the importance of including the management of pediatric SD in any future guidelines in this area. Furthermore, non‐randomized pediatric data in the literature, have not demonstrated any clear choice, duration or route of antibiotic treatment.

A large systemic review of 35 studies and 340 patients showed that ceftriaxone was the most commonly used used IV antibiotic and combination antibiotics were used in 18 out of 32 studies [3]. In the review, antibiotic duration varied from 1 week to 25 weeks for IV and 5 days to 36 months for oral treatment. However, Roversi and colleagues compared pediatric SD patients and a control group of non‐SD osteomyelitis and showed shorter durations in the non‐SD osteomyelitis compared with SD patients (20.5 days vs. 27.5 days) and IV treatment typically less than 2 weeks [6].

In our small study, we found patients who were diagnosed early, could be safely treated with 6 weeks treatment and with a single agent (i.e., IV ceftriaxone followed by oral co‐amoxiclav). A repeat MRI, done before stopping antibiotic treatment, is likely the most accurate biomarker for assessing improvement but our study was too small to confirm this. Future studies should include randomized controlled trials to evaluate the optimal choice and duration of antibiotic treatment.

## Author Contributions

**Sara Farah:** conceptualization, investigation, writing – original draft, methodology, writing – review and editing. **Seilesh Kadambari:** investigation, writing – original draft, methodology, writing – review and editing. **Delane Shingadia:** conceptualization, writing – original draft, methodology, writing – review and editing, investigation.

## Conflicts of Interest

The authors declare no conflicts of interest.

## Transparency Statement

Seilesh Kadambari affirms that this manuscript is an honest, accurate, and transparent account of the study being reported; that no important aspects of the study have been omitted; and that any discrepancies from the study as planned (and, if relevant, registered) have been explained.

## Supporting information

STROBE checklist discitis SUBMITTED 9.

## Data Availability

The authors confirm that the data supporting the findings of this study are available within the article. All patient are data are fully anonymised. The data that support the findings of this study will be available from the corresponding author upon reasonable request.
